# Mn_3_O_4_@ZnO Hybrid Material: An Excellent Photocatalyst for the Degradation of Synthetic Dyes including Methylene Blue, Methyl Orange and Malachite Green

**DOI:** 10.3390/nano12213754

**Published:** 2022-10-26

**Authors:** Benazir Shaikh, Muhammad Ali Bhatti, Aqeel Ahmed Shah, Aneela Tahira, Abdul Karim Shah, Azam Usto, Umair Aftab, Sarah I. Bukhari, Sultan Alshehri, Syed Nizam Uddin Shah Bukhari, Matteo Tonezzer, Brigitte Vigolo, Zaffar Hussain Ibhupoto

**Affiliations:** 1 Institute of Environmental Sciences, University of Sindh, Jamshoro 76080, Pakistan; 2 Wet Chemistry Laboratory, Department of Metallurgical Engineering, NED University of Engineering and Technology, Karachi 75270, Pakistan; 3 Dr. M.A Kazi Institute of Chemistry, University of Sindh, Jamshoro 76080, Pakistan; 4 Department of Chemical Engineering, Dawood University of Engineering and Technology, Karachi 74800, Pakistan; 5 Department of Metallurgy and Materials Engineering, Mehran University of Engineering and Technology, Jamshoro 7680, Pakistan; 6 Department of Pharmaceutics, College of Pharmacy, King Saud University, Riyadh 11451, Saudi Arabia; 7 State Key Laboratory of Organic-Inorganic Composites, Beijing Key Laboratory of Electrochemical Process and Technology for Materials, School of Material Science, Beijing University of Chemical Technology, Beijing 100029, China; 8 Department of Basic Science and Humanities, Dawood University of Engineering and Technology, Karachi 74800, Pakistan; 9 IMEM-CNR, Sede di Trento-FBK, Via alla Cascata 56/C, 38123 Trento, Italy; 10 Institut Jean Lamour, Université de Lorraine, CNRS, IJL, F-54000 Nancy, France

**Keywords:** ZnO, Mn_3_O_4_@ZnO composites, photocatalytic applications

## Abstract

In this study, we synthesized hybrid systems based on manganese oxide@zinc oxide (Mn_3_O_4_@ZnO), using sol gel and hydrothermal methods. The hybrid materials exhibited hierarchical morphologies and structures characterized by the hexagonal phase of ZnO and the tetragonal phase of Mn_3_O_4_. The hybrid materials were tested for degradation of methylene blue (MB), methyl orange (MO), and malachite green (MG) under ultraviolet (UV) light illumination. The aim of this work was to observe the effect of various amounts of Mn_3_O_4_ in enhancing the photocatalytic properties of ZnO-based hybrid structures towards the degradation of MB, MO and MG. The ZnO photocatalyst showed better performance with an increasing amount of Mn_3_O_4_, and the degradation efficiency for the hybrid material containing the maximum amount of Mn_3_O_4_ was found to be 94.59%, 89.99%, and 97.40% for MB, MO and MG, respectively. The improvement in the performance of hybrid materials can be attributed to the high charge separation rate of electron-hole pairs, the co-catalytic role, the large number of catalytic sites, and the synergy for the production of high quantities of oxidizing radicals. The performance obtained from the various Mn_3_O_4_@ZnO hybrid materials suggest that Mn_3_O_4_ can be considered an effective co-catalyst for a wide range of photocatalytic materials such as titanium dioxide, tin oxide, and carbon-based materials, in developing practical hybrid photocatalysts for the degradation of dyes and for wastewater treatment.

## 1. Introduction

Environmental pollution is caused by the extensive use of synthetic dyes, pigments, and other coloring products, in various industries such as food, beverages, cosmetics, pharmaceuticals, clothes and paper [1]. These chemical compounds are characterized by a complicated structure, high stability and highly toxicity to living things. In addition, the presence of dyes in wastewater prevents the transmission of sunlight deep into the water, damaging aquatic life. At the same time, these dyes are harmful to humans by causing breathing problems, vomiting, cancer, allergies, and skin problems [2]. Approximately 700,000 tons of dyes are produced worldwide each year, and many end up in the wastewater of the textile industries, contaminating water resources and aquatic life, as they are very toxic to our environment [3,4]. Among them, malachite green (MG) and methylene blue (MB) are cationic, while methyl orange (MO) is anionic in nature [5,6]. Environmental pollution caused by organic dyes threatens life on land and in water. Therefore, immediate planning and action is needed to eliminate environmental pollution using new technologies, but sadly, to this date it has remained a challenging task.

Currently, the removal and degradation of dyes from wastewater before discharging it from reservoirs is seriously considered, but it still seems a very difficult challenge. There are numerous methods for wastewater treatment including coagulation, electro precipitation, adsorption, evaporation, reverse osmosis, flocculation, oxidation and photodegradation under the illumination of ultraviolet and solar light [7,8]. Many of these methods are complicated, expensive, and have poor dye-removal performance, while photodegradation is a simple, low cost and efficient method of removing organic dyes from wastewater [9,10]. The challenge in photocatalytic degradation is the unavailability of highly efficient yet inexpensive photocatalysts. For this purpose, several photocatalytic materials have been prepared and tested for the degradation of dyes, such as ZnO [11], BiFeO_3_ [12], TiO_2_ [10], Cu_2_O/LDH [13], Chitosan ZnO composite [14], CuCl_2_ + STS, chitosan-g-Poly (acrylamide)/ZnS [15], Chitosan/LaFe_0.8_Cu_0.2_O_3_ and xylan/LaFe_0.8_Cu_0.2_O_3_ [16].

Among these well-known semiconducting materials, ZnO is highly studied for various applications including photocatalysis, chemical sensing and photovoltaics, due to its tunable band gap (3.37 eV) and high exciton binding energy (60 meV) [17,18,19,20]. The role of the nanostructured morphology of ZnO is intensively investigated, and therefore different morphologies of ZnO are reported, such as 3D hollow spheres, 1D nanorods and nanowires and 2D nanobelts and nanosheets [21,22,23,24]. The advantageous features of these morphologies such as swift mass transfer, hierarchical structures and high surface area have been utilized for different applications, such as chemical sensors, lithium-ion batteries and photocatalysis [25,26]. However, the growing concern for the environment requires the development of nanostructured materials without, or with a minimum use of, harmful chemicals [27,28,29]. Therefore, it is essential to synthesize ZnO-based nanostructures with maximum photocatalytic efficacy in an eco-friendly manner. Manganic oxide (Mn_3_O_4_) is an emerging *p*-type semiconductor with significant photocatalytic properties [30,31]. Previously, Mn_3_O_4_ has demonstrated very good performance in photocatalytic applications, especially in combination with other metal oxides [32,33,34,35,36,37,38,39,40]. These studies suggest that Mn_3_O_4_ can be used as a potential counterpart for photocatalytic materials and can enhance the catalytic activity of those materials through the synergetic effect of an enhanced surface area for hosting photocatalytic materials. ZnO has many positive aspects, such as high quantum efficiency, rich defect chemistry, and cost effectiveness, and is therefore widely used for photocatalysis [39,40]. The synthesis of hierarchical nanostructures of hybrid material based on Mn_3_O_4_@ZnO with improved electron-hole pairs separation and high density of electrons in the conduction band of ZnO, could lead to more efficient photodegradation of MB, MO and MG under ultraviolet light illumination. A hybrid system based on Mn_3_O_4_@ZnO for efficient degradation of MB, MO and MG has not yet been reported in the exiting scientific literature.

In this study, we prepared the Mn_3_O_4_@ZnO hybrid material using the sol gel method followed by the hydrothermal method. The morphology, crystalline structure and chemical composition were studied with different analytical techniques such as SEM, XRD and EDS. The optimized Mn_3_O_4_@ZnO hybrid system revealed excellent photoactivity in the degradation of MB, MO and MG under UV light illumination.

## 2. Materials and Methods

### 2.1. Materials Used

Potassium permanganate (KMnO_4_) (99.0%), glycerol (99.5%), zinc acetate dihydrate (99.99%), 33% aqueous ammonia, ethanol (99.98%), sodium hydroxide (98%), hydrochloric acid (37%), methyl orange (85%), methylene blue (70%), and malachite green (97%) were purchased from Sigma Aldrich, Karachi, Pakistan. The chemicals used were of a high analytical grade, and were used without pretreatment. All desired concentrations of each reagent were prepared in deionized water.

### 2.2. Synthesis of Z Mn_3_O_4_@ZnO Hybrid Materials Using Sol Gel and Hydrothermal Methods

**Scheme 1:**
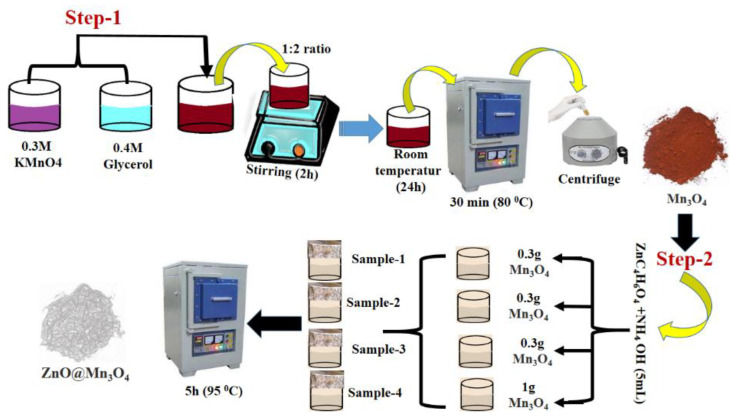
Stepwise synthesis of Mn_3_O_4_@ZnO hybrid materials using sol gel and hydrothermal methods.

The synthesis of the nanostructured hybrid material was carried out in two steps. The Mn_3_O_4_ nanostructures were prepared using the sol gel method, and the typical synthesis process is described below. The Mn_3_O_4_ material was reproduced according to the work reported in [41]. Precursor solutions of 0.4 M glycerol and 0.3 M potassium permanganate were prepared. The glycerol and potassium permanganate solutions were then mixed in a ratio of 1:2 by volume, stirred for 2 h, and finally kept for 24 h without disturbance, to obtain the desired gel at room temperature. Afterwards, the gel was heated at 80 °C for 30 min, the product precipitates were obtained, and centrifugation was used to collect the fine brown-colored Mn_3_O_4_ powder. Meanwhile, the precursor ZnO was prepared by mixing 0.1 M of zinc acetate dihydrate and 5 mL of 33% aqueous ammonia solution in 100 mL of deionized water. Different amounts of Mn_3_O_4_ were used for the deposition of ZnO in the hydrothermal method: 0.3, 0.5, 0.75 and 1.0 g of precipitated Mn_3_O_4_ were dissolved in four beakers containing 200 mL of a precursor solution of ZnO, in order to develop hybrid Mn_3_O_4_@ZnO systems. The materials were labeled as sample 1, sample 2, sample 3, and sample 4, respectively. A fifth beaker contained only the ZnO precursor solution, and the pristine ZnO sample was used as a comparison. After being mixed, the solutions were covered very tightly with aluminum foil, and left for hydrothermal reaction in a preheated electric oven for 5 h at 95 °C. After completion of the reaction, the beakers were cooled down to room temperature and the product was collected on filter paper and washed several times with ethanol followed by deionized water. Finally, the product was dried at 60 °C overnight. The experimental layout is given in Scheme 1.

### 2.3. Characterization of Morphology, Crystalline Structure, and Chemical Composition of the Materials

Scanning electron microscopy (Jeol, JSM-6380 A, Tokyo, Japan)was used at 10 kV to evaluate the morphology of the prepared nanostructured materials. Powder X-ray diffraction (Miniflex 600, Rigaku, Austin, TX, USA) was employed at 45 mA and 45 kV to identify the crystal structure and phase of the nanostructured materials. Energy dispersive spectroscopy (Jeol, JSM-6380 A) was used to quantify the elemental composition of each synthesized material. Following this, UV-visible spectrophotometry (PE Lamda365, Waltham, MA, USA) was employed to record absorption spectra during dye degradation under UV light illumination. The UV light source consisted of six UV tubes with a wavelength of 365 nm.

In this study, we studied the performance of hybrid materials for the degradation of various dyes (methylene blue, methyl orange, and malachite green), using 5 mg of each catalyst nanomaterial, and a fixed concentration for each dye (1.87 × 10^−5^ M MB, 2.44 × 10^−5^ M MO, and 5.48 × 10^−5^ M MG). Before irradiation with UV light, the dye solution was stirred for 30 min, in order to optimize its homogeneity.

The % degradation efficiency of each dye was estimated from the following equation:

(1) $$\mathrm{dye}\;\mathrm{removal}\;\%=\frac{C_{o}-C_{t}}{C_{o}}\times 100$$

where *C_o_* and *C_t_* are the concentrations of each dye before and after degradation, respectively.

## 3. Results and Discussion

### 3.1. Characterization of Morphology, Crystalline Structure, and Chemical Composition of the Mn_3_O_4_@ZnO Hybrid Materials

**Figure 1:**
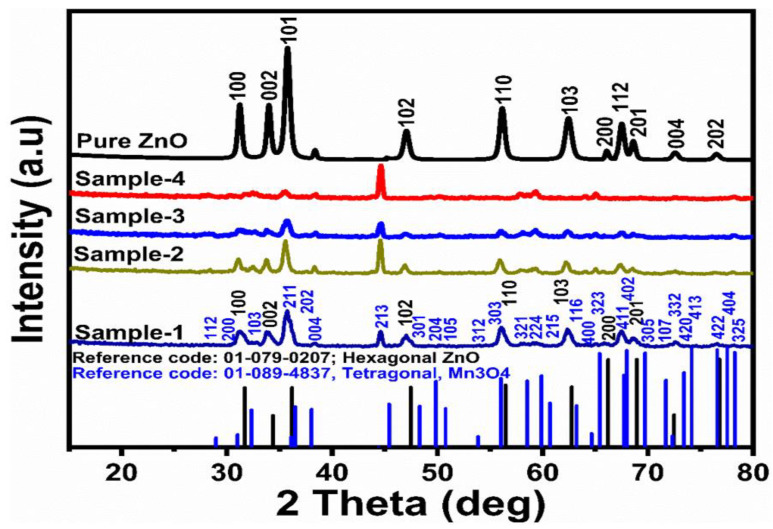
XRD patterns of pristine ZnO and Mn_3_O_4_@ZnO hybrids with different amounts of Mn_3_O_4_.

Figure 1 shows the XRD patterns of the different samples with increasing amounts of Mn_3_O_4_. The pattern obtained from the pure ZnO sample shows all the characteristic peaks of the material, as can be seen from the comparison with the black reference at the bottom of the figure (JCPDS sheet: 01-079-0207). In detail, the peaks present at 31.66°, 34.29°, 36.23°, 47.48°, 56.60°, 62.77° and 67.85° can be attributed to the planes (100), (002), (101), (102), (110), (103) and (200) of the hexagonal structure of the ZnO wurtzite. The patterns obtained from the hybrid samples also show the peaks from ZnO, but their relative intensity decreases, while that of the peaks from the tetragonal phase of Mn_3_O_4_ increases (JCPDS sheet: 00-003-1041, in blue at the bottom of the figure). The XRD patterns shown in Figure 1 demonstrate that the Mn_3_O_4_@ZnO hybrid was successfully prepared. The nanomaterial is composed of ZnO and Mn_3_O_4_, while no amorphous phases or impurities were observed.

**Figure 2:**
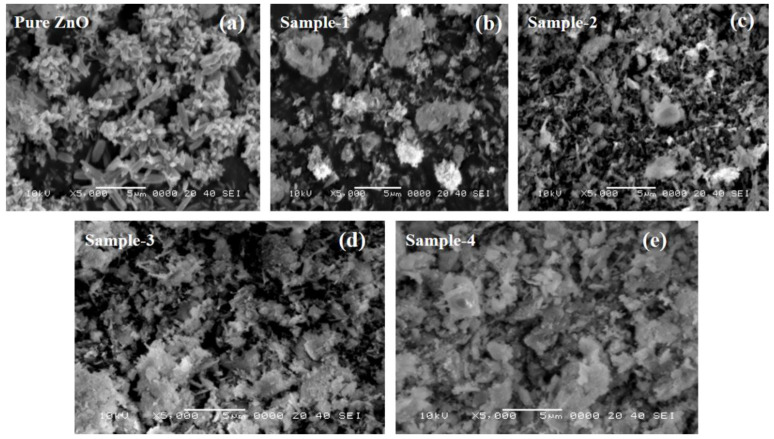
SEM images of (**a**) pristine ZnO and (**b**–**e**) Mn_3_O_4_@ZnO materials synthesized by sol-gel method.

**Figure 3:**
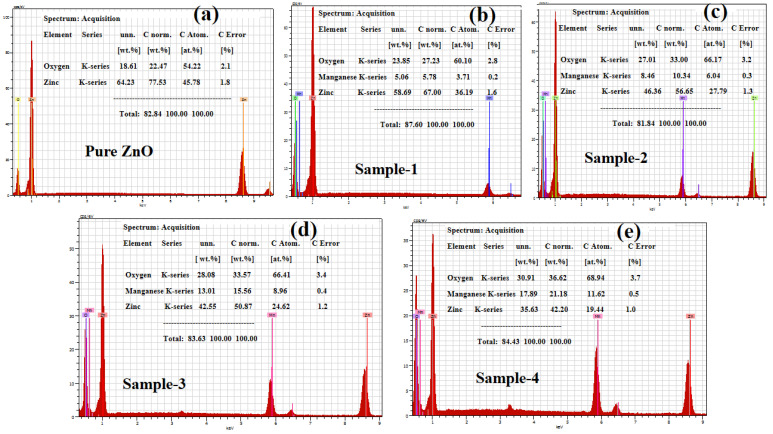
(**a**) EDX analysis of (**a**) pristine ZnO and (**b**–**e**) Mn_3_O_4_@ZnO material synthesized by Sol-gel method.

The SEM images in Figure 2 show the morphology of pure ZnO and of the Mn_3_O_4_@ZnO hybrids. Pure ZnO appears to be made up of flower-like structures composed of nanorods with a diameter of 200–500 nm (Figure 2a). Conversely, the morphology of hybrid nanomaterials appears to change towards very thin sheets as the Mn_3_O_4_ content increases (Figure 2b–e). The morphology of all the samples is very homogeneous, indicating a good mixing of ZnO and Mn_3_O_4_ during the hydrothermal process. The elemental composition of the materials was studied by energy dispersion X-ray spectroscopy, and the spectra are shown in Figure 3. The spectrum of pure ZnO (Figure 3a) shows only peaks relative to Zn and O, while the other samples show the presence of Zn, O and Mn (Figure 3b–e). The atomic percentage of manganese increases from Sample 1 to Sample 4, as expected.

### 3.2. Photocatalytic Degradation of MB, MO and MG onto Different Mn_3_O_4_@ZnO Hybrid Systems

The photodegradation of MB, MO and MG in aqueous solution using pure ZnO and the different hybrid systems ZnO@Mn_3_O_4_ was studied under UV light illumination.

#### 3.2.1. Methylene Blue

**Figure 4:**
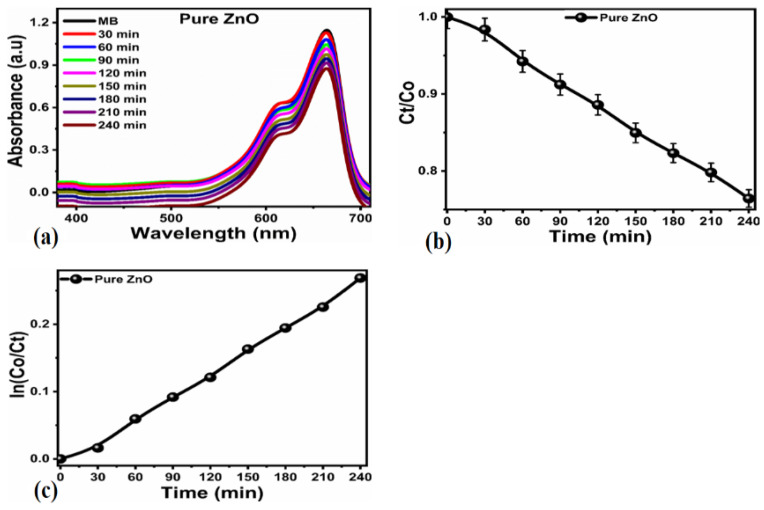
Degradation over time (240 min) of a solution of MB of concentration 1.87 × 10^−5^ M under irradiation with UV light in the presence of 5 mg of pristine ZnO. (**a**) UV-vis absorption spectra during dye degradation, (**b**) kinetics *C_t_*/*C_o_* irradiation time, (**c**) Ln*C_t_*/*C_o_* plot of pure ZnO over MB.

Figure 4 shows the absorption spectra of pure ZnO during MB degradation over time under UV light illumination. A solution of MB with a concentration of 1.87 × 10^−5^ M was used, to which 5 mg of catalyst was added. The spectra show a typical absorption peak around 663 nm which slowly decreases over time, indicating that the degradation rate is limited and therefore takes a long time. This suggests that pure ZnO exhibits a low density of catalytic sites and a high rate of recombination of the electron-hole pair; therefore, the release of oxidizing agents under illumination is limited, thus leading to poor MB degradation performance. The degradation kinetics was also studied as shown in Figure 4b,c. The reaction rate of MB degradation was evaluated by monitoring the change in concentration *C_t_* over time from the initial concentration *C_o_*, as shown in Figure 4b, and the linear fitting revealed the fitting error of less than 5%. Clearly, the photoactivity of pure ZnO is highly correlated to the illumination time. The rate constant (*K*) of the photodegradation process was also calculated using the first order reaction equation [42]:

*
K_t_*
= Ln(*C_o_*/*C_t_*)

The reaction kinetics was also studied as Ln(*C_o_*/*C_t_*) over the illumination time, and is shown in Figure 4c. The rate constant for MB degradation using pure ZnO was found to be 1.14 × 10^−3^ min, confirming that the reaction follows first-order kinetics.

**Figure 5:**
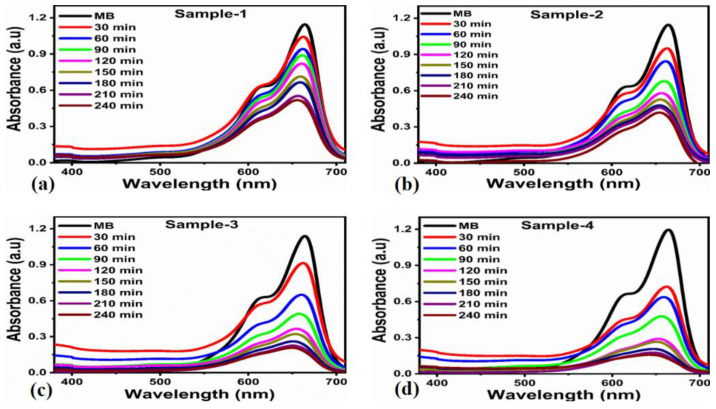
(**a**–**d**) UV-Vis absorption spectra of the MB solution at concentration of 1.87 × 10^−5^ M, using 5 mg photocatalyst dose of hybrid materials during photodegradation under UV light illumination, using samples 1–4 as photocatalysts.

**Figure 6:**
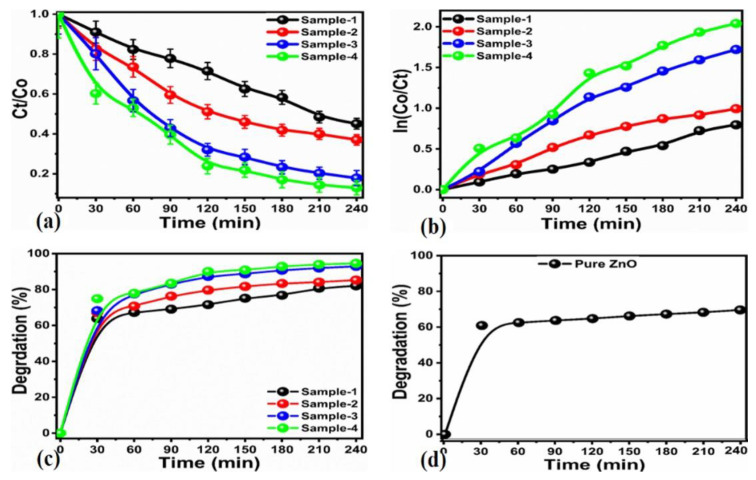
Degradation kinetics (**a**) kinetics *C_t_*/*C_o_* irradiation time, (**b**) Ln*C_t_*/*C_o_* plot, (**c**) percent degradation of MB, (**d**) degradation percentage of pure ZnO over MB.

The degradation of MB was also performed using the various samples, in order to verify how the degradation rate is affected by the composition of the hybrid material, and the spectra are shown in Figure 5a–d. The four panels show that all samples work better as photocatalysts than pure ZnO. Furthermore, comparing the decrease of the absorption spectrum over time in the four panels, it is clear that a higher amount of Mn_3_O_4_ in the hybrid material greatly improves the degradation of MB. This trend could be attributed to the role of Mn_3_O_4_ in separating the charges of the electron-hole pairs, resulting in the production of a high amount of oxidizing radicals that play an important role in driving the degradation of MB in aqueous solution. The reaction kinetics was investigated for the hybrid samples of Mn_3_O_4_@ZnO, and the ratio between the concentrations of MB at a certain time *C_t_* to the initial concentration *C_o_* is shown in Figure 6a. The degradation of MB on hybrid materials clearly depends on the illumination time, and the linear fitting revealed a fitting error of less than 5%. The reaction kinetics in terms of order of reaction was studied by calculating Ln(*C_o_*/*C_t_*) over time (Figure 6b), and it was found that the rate constant was characterized by first-order kinetics. The rate constant (*K*) values for samples 1, 2, 3 and 4 were estimated to be 3.33 × 10^−3^ min, 4.19 × 10^−3^ min, 7.33 × 10^−3^ min, and 8.51 × 10^−3^ min, respectively. These values of rate constant suggest that a higher amount of Mn_3_O_4_ in the hybrid material significantly improves the degradation kinetics of MB in aqueous solution under UV light illumination. The degradation efficiency was calculated from the absorbance spectra and plotted over time for each hybrid system, in Figure 6c. The hybrid Mn_3_O_4_@ZnO system with the highest amount of Mn_3_O_4_ (sample 4) was the most efficient, with a degradation efficiency of 94.59% for MB. The degradation efficiency of the other hybrid samples 1, 2, and 3 was 82.07%, 85.28%, and 92.91%, respectively. The degradation efficiency of pure ZnO photocatalyst was found to be 69.60%, as shown in Figure 6d, indicating that the performance of pure ZnO is not sufficient, and the photocatalytic properties of the proposed hybrid system are improved.

#### 3.2.2. Methyl Orange

**Figure 7:**
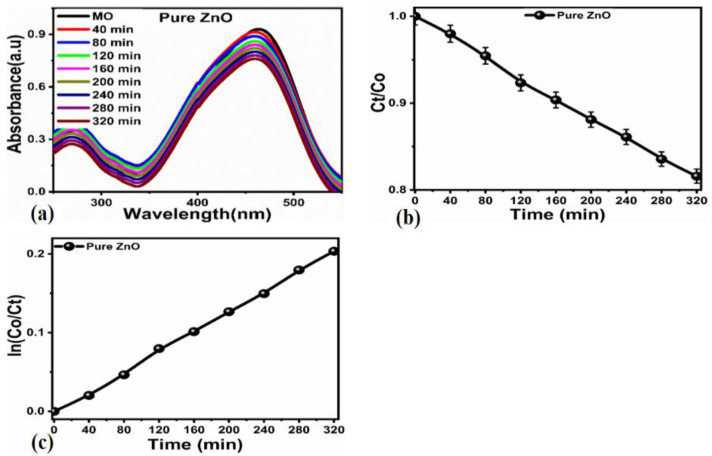
Degradation over time (320 min) of a solution of MO of concentration 2.44 × 10^−5^ M under irradiation with UV light, in the presence of 5 mg of pristine ZnO. (**a**) Time change of the UV-vis spectrum, (**b**) kinetics *C_t_*/*C_o_* irradiation time, (**c**) Ln*C_t_*/*C_o_* plot of pure ZnO over MO.

**Figure 8:**
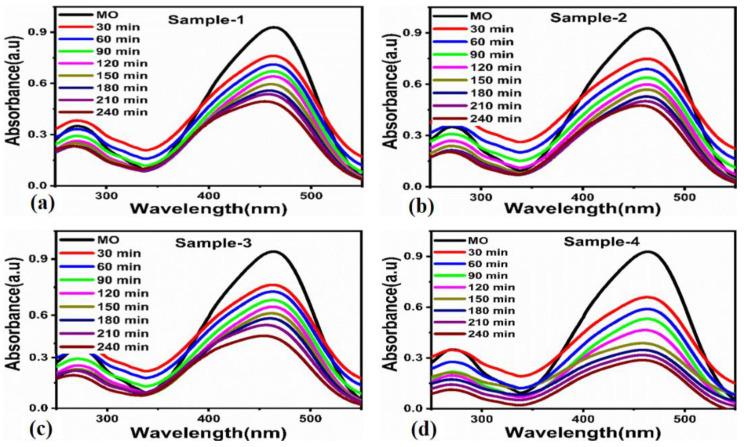
(**a**–**d**) UV-Vis absorption spectra MO solution at concentration of 2.44 × 10^−5^ M during photodegradation under UV light illumination, using 5 mg dose of samples 1–4 as hybrid photocatalysts.

**Figure 9:**
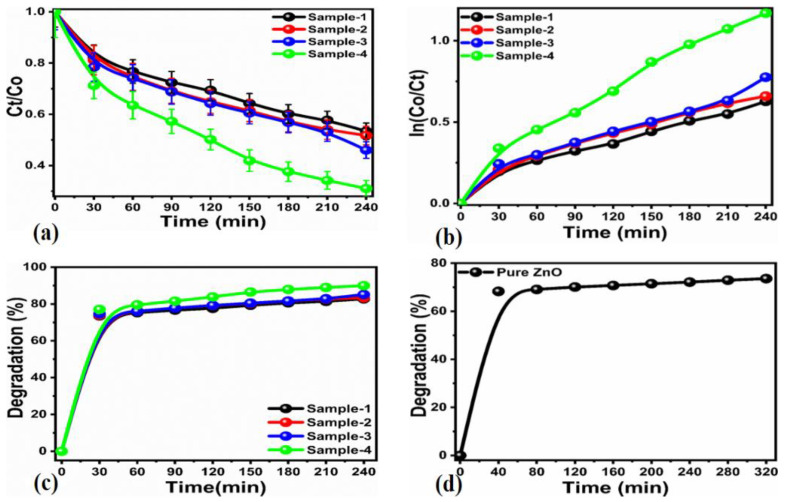
Degradation kinetics (**a**) kinetics *C_t_*/*C_o_* irradiation time, (**b**) Ln*C_t_*/*C_o_* plot, (**c**) percent degradation of MO, (**d**) degradation percentage of pure ZnO over MO.

The photodegradation of methyl orange was investigated with a solution with a concentration of 2.44 × 10^−5^ M and 5 mg of photocatalyst under UV light illumination. In the case of pure ZnO (Figure 7), the degradation was found to be very poor, due to the stable nature of the material, limited number of active sites, and the rapid rate of charge recombination. The absorption trend is very slow, which means that the reaction rate is associated with a high activation energy using pure ZnO as photocatalyst. Poor performance in the degradation of MO by pure ZnO indicates that it is not a suitable material for this application. The reaction kinetics was studied by evaluating the concentration of MO over time, *C_t_*, with respect to the initial concentration, *C_o_*, as shown in Figure 7a, and indicates that the linear fitting revealed a fitting error of less than 5%. The plot in Figure 7b shows that the degradation is linear, with respect to the irradiation time with UV light. The reaction kinetics with respect to UV light irradiation time turned out to be of the first order, and the rate constant was calculated to be 6.44 × 10^−4^ min^−1^, as shown in Figure 7c. The degradation efficiency for MO was calculated from the absorption spectra of MO over a time of 320 min, and the calculated value was 73.56%, as shown in Figure 7d. The four different hybrid samples were tested for the degradation of MO, using a solution with a concentration of 2.44 × 10^−5^ M and 5 mg of each hybrid catalyst. The degradation experiments lasted 240 min, and an adsorption spectrum was acquired every 30 min, as shown in Figure 8. Comparing the plots in Figure 8 with that in Figure 7a, it is clear, albeit qualitatively, that the degradation is in all cases better than that of pure ZnO. This indicates that the presence of Mn_3_O_4_ in the ZnO@Mn_3_O_4_ hybrid material increases the density of active sites and decreases the charge recombination rate of electron-hole pairs. In fact, the degradation of MO increases as the quantity of Mn_3_O_4_ present in the hybrid photocatalyst increases, i.e., from sample 1 to sample 4. The degradation kinetics of MO was also studied by evaluating how the MO concentration over time, *C_t_*, varies with respect to the initial concentration, *C_o_*, (Figure 9a), and the linear fitting revealed a fitting error of less than 5%. The Ln of the ratio is instead shown in Figure 9b. As can be seen in both panels, the degradation rate strongly depends on the irradiation time with UV light. The reaction kinetics of the four hybrid photocatalysts are of the first order, and the rate constant values obtained for samples 1, 2, 3 and 4 are respectively 2.32 × 10^−3^ min, 2.50 × 10^−3^ min, 2.74 × 10^−3^ min and 4.58 × 10^−3^ min. The percent degradation efficiency of samples 1, 2, 3 and 4 was calculated and found to be 82.78%, 83.38%, 85.12%, and 89.99%, respectively, as shown in Figure 9c. It is therefore clear that the degradation efficiency for MO increases as the quantity of Mn_3_O_4_ in the hybrid material increases. The degradation efficiency for MO was calculated from the absorption spectra of MO over a time of 320 min, and the calculated value was 73.56%, as shown in Figure 9d.

#### 3.2.3. Malachite Green

**Figure 10:**
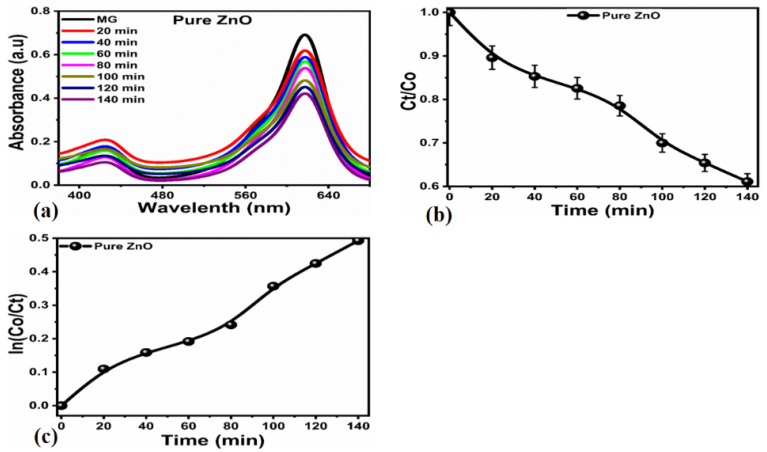
Degradation over time (140 min) of a solution of MG of concentration 5.48 × 10^−5^ M under irradiation with UV light in the presence of 5 mg of pristine ZnO. (**a**) Time change of the UV-vis spectrum, (**b**) kinetics *C_t_*/*C_o_* irradiation time, (**c**) Ln*C_t_*/*C_o_* plot of pure ZnO over MG.

Considering the high toxicity of MG to aquatic life and human health, pure ZnO and Mn_3_O_4_@ZnO hybrid materials were tested for the degradation of MG under UV light illumination. In the experiments, a solution of MG with a concentration of 5.48 × 10^−5^ M and 5 mg of catalyst were used. Figure 10 shows the maximum absorption peak of MG at 617 nm and its evolution over time in the presence of pure ZnO. The adsorption peak decreases, confirming the catalytic activity; however, the degradation process is relatively slow, and the efficiency is not very high, due to the wide band gap of pure ZnO, its fast charge recombination rate and its low density of catalytic sites. The reaction rate of MG with pure ZnO was studied quantitatively by plotting the ratio between the concentration over time, *C_t_*, and the initial concentration, *C_o_*, and it is clear that the degradation of MG strongly depends on the time of irradiation with UV light (Figure 10b). Furthermore, plotting Ln*C_o_*/*C_t_* as in Figure 10c, it can be seen that the reaction kinetics are of the first order. The percent degradation efficiency was calculated, obtaining a value of 85.37%, as shown in Figure 10d. The performance of pure ZnO was therefore unsatisfactory, demonstrating that the material must be functionalized to efficiently degrade MG under UV light illumination.

**Figure 11:**
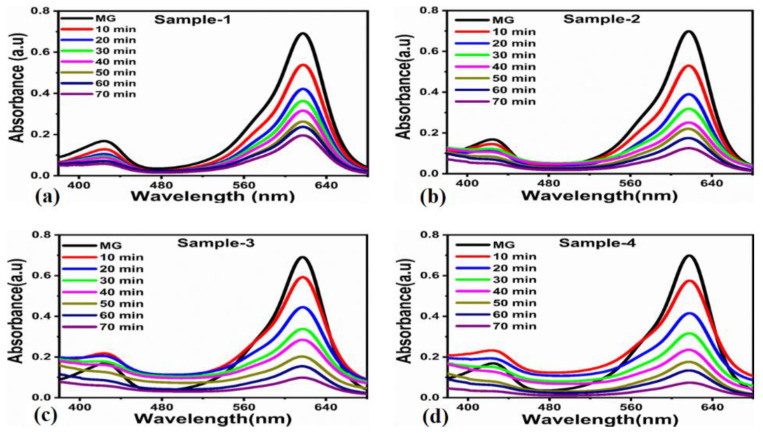
(**a**–**d**) change in the UV-Vis spectrum of the MG solution at concentration of 5.48 × 10^−5^ M during photodegradation under UV light irradiation, using 5 mg dose of samples 1–4 as photocatalysts.

In this way, the hybrid materials Mn_3_O_4_@ZnO were also tested, with their absorption spectra over time shown in Figure 11. All materials functionalized with Mn_3_O_4_ proved to be highly efficient for the degradation of MG, as can be seen from the strong decrease of the absorption peak at 617 nm. Note that the degradation experiment in this case only lasted 70 min. Comparing the four panels (a–d), it can be seen that a greater quantity of Mn_3_O_4_ increases degradation efficiency. The excellent performance of the hybrid materials can therefore be reasonably attributed to the functionalization with Mn_3_O_4_, which acts as a co-catalyst and lowers the charge recombination rate of the electron-hole pairs. The synergy of the two materials allows a faster production of oxidizing radicals, which increases the degradation rate of MG in aqueous solution [38,39,40]. The synergy of high surface area due to the nanostructured nature of Mn_3_O_4_ and the higher exposure of catalytic sites from ZnO allowed the hybrid material to behave effectively for the degradation of various synthetic dyes [38,39,40].

**Figure 12:**
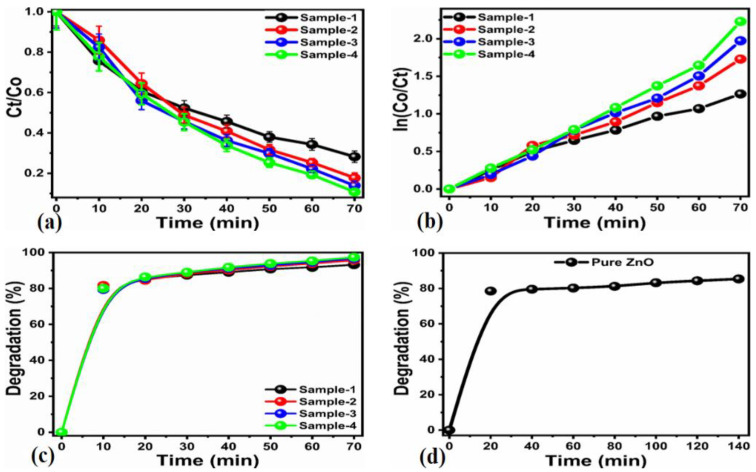
Degradation kinetics (**a**) kinetics *C_t_*/*C_o_* irradiation time, (**b**) Ln*C_t_*/*C_o_* plot, (**c**) percent degradation of MG, (**d**) degradation percentage of pure ZnO over MG.

The reaction rate was quantitatively assessed in terms of the change in MG concentration over time, *C_t_*, with respect to its initial concentration, *C_o_*, as shown in Figure 12a, and suggested a fitting error of less than 5%. The curves show that the degradation of MG in aqueous solution depends on the time of illumination with UV light. The kinetics of the degradation of MG is first order for all hybrid photocatalysts Mn_3_O_4_@ZnO, and the rate constants for samples 1, 2, 3 and 4 are calculated in 1.72 × 10^−2^ min, 2.38 × 10^−2^ min, 2.73 × 10^−2^ min, and 3.01 × 10^−2^ min, respectively, as shown in Figure 12b. The degradation efficiency for the four hybrid materials (samples 1, 2, 3 and 4) was calculated as 93.22%, 95.71%, 96.67%, and 97.40%, respectively, as shown in Figure 12c. The percent degradation efficiency of pure ZnO was calculated, and obtained a value of 85.37%, as shown in Figure 12d.

Overall, the degradation measurements of MB, MO and MG dyes demonstrated that Mn_3_O_4_@ZnO hybrid nanomaterials are excellent photocatalysts. The photocatalytic activity of the hybrid material with the maximum amount of Mn_3_O_4_ was the best for all three dyes, reaching almost 100% for MG. The performance of the various Mn_3_O_4_@ZnO hybrid materials for the degradation of MB, MO and MG is summarized in Table 1. The activity of the Mn_3_O_4_@ZnO hybrid system towards MB, MO and MG was compared with the recently reported works, as given in Table 2, and it is obvious that the presented photocatalyst is a potential and alternative candidate to utilize within practical wastewater treatment. The advantageous features are its simplicity, significant efficiency, and multiple dye-degradation aspects. However, the understanding of improved performance has to be further characterized by different analytical techniques such as X-ray photoelectron spectroscopy, high resolution transmission electron microscopy, and photoluminescence spectroscopy.

## 4. Conclusions

In this research work, we used sol gel and hydrothermal methods to synthesize different nanostructured materials based on pristine ZnO and functionalized with different amounts of Mn_3_O_4_. The morphology turned out to be hierarchical and composed of thin nanosheets, the composition containing only Zn, O and Mn, and the structure composed of the hexagonal phases of ZnO and tetragonal of Mn_3_O_4_. The degradation performance of all tested dyes (MB, MO and MG) increased as the amount of Mn_3_O_4_ in the Mn_3_O_4_@ZnO hybrid photocatalyst increased. The material with the highest amount of Mn_3_O_4_ (1 g) showed excellent degradation properties for all dyes: 94.59%, 89.99% and 97.40% for MB, MO and MG, respectively. The degradation for all dyes followed first-order kinetics. The rate constant values for the best catalyst were 8.51 × 10^−3^ min for MB, 4.58 × 10^−3^ min for MO, and 3.01 × 10^−2^ min for MG. The improved performance of hybrid materials can be attributed to the synergy between ZnO and Mn_3_O_4_, which leads to a high charge separation rate, a high density of catalytic sites, and the generation of a large amount of oxidizing reactive species. These improvements suggest that Mn_3_O_4_ can be used as a dopant to increase the photocatalytic properties of other metal oxides such as TiO_2_, SnO_2_ and carbon-based materials.

## Figures and Tables

**Table 1:** Summary of obtained results for the photodegradation of various dyes using different Mn_3_O_4_@ZnO hybrid materials in comparison with pure ZnO.

Sample Name	Dye	Degradation %	Time (min)	Rate Constant (*K*)
Pure ZnO	MB	69.60%	240 min	1.14 × 10^−3^ min
	MO	73.56%	320 min	6.43951 × 10^−4^ min
	MG	85.37%	140 min	3.37 × 10^−3^ min
Sample-1	MB	82.07%	240 min	3.33 × 10^−3^ min
	MO	82.78%	240 min	2.32 × 10^−3^ min
	MG	93.22%	70 min	1.72 × 10^−2^ min
Sample-2	MB	85.28%	240 min	4.19 × 10^−3^ min
	MO	83.38%	240 min	2.50 × 10^−3^ min
	MG	95.71%	70 min	2.38 × 10^−2^ min
Sample-3	MB	92.91%	240 min	7.33 × 10^−3^ min
	MO	85.12%	240 min	2.74 × 10^−3^ min
	MG	96.67%	70 min	2.73 × 10^−2^ min
Sample-4	MB	94.59%	240 min	8.51 × 10^−3^ min
	MO	89.99%	240 min	4.58 × 10^−3^ min
	MG	97.40%	70 min	3.01 × 10^−2^ min

**Table 2:** Comparison table of previously reported photocatalysts and their degradation efficiency.

Catalyst	Light Source	Pollutant	Time	Degradation %	Reference
FNZP/PAM	Sunlight	MB/MG	120	96.13%/97.56%	[43]
AFe_2_O_4_ nanoparticles (A = Mg, Zn, Mn)	UV light	MB	120	99.92%	[44]
Banyan leaves	-	MG/MB/MO	20	98%, 96%/23.80%	[45]
MnTiO_3_	Sunlight	MB	240	75%	[46]
Mn_3_O_4_/10%GO NC	UV lamp	MB	180	60%	[47]
Mn_3_O_4_	UV light	Congo red	20	38%	[48]
Mn_3_O_4_/GO composite	LED Lamp	MO	240	95%	[49]
rGO/Mn_3_O_4_ nanocomposites	Sunlight	MB	60	73–96%	[50]
Mn_3_O_4_	HG lamp	MB	240	21%	[51]
Mn_3_O_4_/ZnO/Eu_2_O_3_	Sun light	MB, MO	150	98%, 96%	[52]
Ag–Mn_3_O_4_	Visible light	MB	120	90%	[53]
Mn_3_O_4_	UV light	MB	40	68%	[54]
Mn_3_O_4_@ZnO	UV light	MG, MO, MG	70, 240, 240	97.40%, 94.59%, 89.99%	Present work

## Data Availability

Not Applicable.
